# Further accelerating biologics development from DNA to IND: the journey from COVID-19 to non-COVID-19 programs

**DOI:** 10.1093/abt/tbae001

**Published:** 2024-01-24

**Authors:** Kee Wee Tan, Pengfei Ji, Hang Zhou, Sam Zhang, Weichang Zhou

**Affiliations:** Cell Line Development, WuXi Biologics, 288 Fute Zhong Road, Waigaoqiao Free Trade Zone, Shanghai 200131, China; Cell Line Development, WuXi Biologics, 288 Fute Zhong Road, Waigaoqiao Free Trade Zone, Shanghai 200131, China; Bioprocess Research & Development, WuXi Biologics, 288 Fute Zhong Road, Waigaoqiao Free Trade Zone, Shanghai 200131, China; Cell Line Development, WuXi Biologics, 288 Fute Zhong Road, Waigaoqiao Free Trade Zone, Shanghai 200131, China; Biologics Development, WuXi Biologics, 288 Fute Zhong Road, Waigaoqiao Free Trade Zone, Shanghai 200131, China

**Keywords:** CMC for biologics, COVID-19, mammalian cell line development, neutralizing antibody, speed to clinic

## Abstract

The Coronavirus Disease (COVID‐19) pandemic has spurred adoption of revolutionary initiatives by regulatory agencies and pharmaceutical industry worldwide to deliver therapeutic COVID-19 antibodies to patients at unprecedented speed. Among these, timeline of chemistry, manufacturing and control (CMC), which involves process development and manufacturing activities critical for the assurance of product quality and consistency before first-in-human clinical trials, was greatly reduced from typically 12–15 months (using clonal materials) to approximately 3 months (using non-clonal materials) in multiple cases. In this perspective, we briefly review the acceleration approaches published for therapeutic COVID-19 antibodies and subsequently discuss the applicability of these approaches to achieve investigational new drug (IND) timelines of ≤10 months in over 60 COVID-19 and non-COVID-19 programs performed at WuXi Biologics. We are of the view that, with demonstrated product quality and consistency, innovative approaches used for COVID-19 can be widely applied in all disease areas for greater speed to clinic.

## Teaser

Statement of Significance: The COVID-19 pandemic has propelled unprecedented speed to clinic for neutralizing COVID-19 monoclonal antibodies with demonstrated product quality and consistency. Innovative acceleration strategies used in COVID-19 programs can be considered and holistically applied in biologics development of all disease areas to further shorten CMC timeline.

## INTRODUCTION

Since its emergence in November 2019, Coronavirus Disease (COVID-19) has resulted in over 770 million recorded cases with nearly 7 million fatalities globally as of October 2023 [1]. At the forefront of combating the coronavirus designated as SARS-CoV-2, pharmaceutical industry and regulatory agencies worldwide have taken extraordinary measures in expediting development and approval of vaccines and treatments. Among these, neutralizing monoclonal antibody (mAb) is a major treatment option, especially for patients who are at high risk for progression to severe COVID-19. To date, approved COVID-19 antibodies include REGEN-COV (casirivimab + imdevimab) by Regeneron, JS016/LY-CoV016 (etesevimab) by Lilly and Junshi (TopAlliance), bamlanivimab + etesevimab by Lilly, Xevudy (sotrovimab) by GSK and Vir, EVUSHELD (tixagevimab + cilgavimab) by AstraZeneca and bebtelovimab by Lilly in the USA [2]; amubarvimab + romlusevimab by Brii Biosciences in China [3]; and Regkirona (regdanvimab) by Celltrion in Korea [4]. Many of these COVID-19 antibodies were granted emergency use authorization (EUA) or conditional marketing authorization (CMA) at trailblazing speed, although some had since been withdrawn due to lack of efficacy against evolved SARS-CoV-2 subvariants [5, 6]. The pandemic has highlighted the urgent need to continuously develop and introduce effective treatments with high agility against life-threatening diseases.

Chemistry, manufacturing and control (CMC) encompasses process development and manufacturing activities crucial for the assurance of product quality and consistency before an investigational new drug (IND) application can be submitted for the approval for first-in-human clinical trial. During the COVID-19 emergency, effective compression of the CMC timeline has been demonstrated to be of utmost importance for the rapid and successful launch of neutralizing COVID-19 mAbs. For a comprehensive outlook on the various CMC aspects that were innovated and accelerated during COVID-19, readers are directed to recent, excellent reviews by Kelley *et al.* [6] and Broly *et al.* [5]. In this perspective, we briefly revisit CMC acceleration strategies employed during COVID-19, with a general focus on cell line development, for which we have reported various acceleration strategies previously [7, 8]. We subsequently share insights from developing more than 30 COVID-19 antibodies at WuXi Biologics and further report the use of these acceleration approaches in more than 30 non-COVID-19 programs. We believe the invaluable experiences from developing COVID-19 antibodies will contribute in reshaping and reforming future biologics development and manufacturing timeline.

## OVERVIEW OF ACCELERATION STRATEGIES FOR COVID-19 MABS

Acceleration strategies reported by pharmaceutical companies worldwide to swiftly deliver COVID-19 mAbs to clinics are summarized in Table 1. Of these, the most striking feature is the use of non-clonal cell pools (stable pools) to generate GMP-grade materials for first-in-human clinical trials. As the main innovation enabling a CMC timeline from DNA to IND application within 3 months, this strategy is definitively described by almost all companies listed in Table 1. Transfected stable pools were generally scaled up to 2000 L for the material generation for phase I clinical trials and even through phase II clinical trials as in a case publicly disclosed by Lilly [9]. This strategy requires the entire CMC campaign, from cell transfection to drug product filling, to be executed under GMP conditions to comply with regulatory demands for clinical entry [7, 8]. Alternatively, the research cell bank (RCB) of stable pool generated under GMP conditions can be used [5]. The successful use of non-clonal materials for first-in-human studies, notwithstanding the exceptional endorsement by regulatory agencies under pandemic emergency, lies in the following cumulative evidence and confidence of their applications over the years. Firstly, stable pools have been used for toxicology studies for a number of years with demonstrated product comparability. This “pool-for-tox” strategy allows the accelerated initiation of IND-enabling toxicology studies by 2–4 months [10–16]. Secondly, improved technologies in mammalian expression system has ensured sufficiently high productivity of the pools, without the need for screening a high-producing clone. For instance, the use of transposon-based or retroviral vector-based transgene integration methods (Table 1 and [5]) have enabled the production of stable pool with high titer. Thirdly, a clonal candidate with comparable product quality attributes is clearly achievable from the parental pool, both theoretically and practically as evidenced from the pool-for-tox strategy and the multiple reports listed in Table 1. Thinking one step further for the expedited provision of clinical material, the use of transient expression material has been proposed [17]. However, several issues remain to be addressed, including comparability evidence for transient materials and the challenges associated with providing sufficient plasmid and transfection reagent for large-scale manufacturing [5, 17].

**Table 1:** Acceleration strategies reported for the development of neutralizing COVID-19 mAbs

Company	Reported timeline	Primary acceleration strategies	Reference
AstraZeneca	▪ Initiation of commercial manufacturing in less than 6 months from lead selection (tixagevimab + cilgavimab/AZD7442/EVUSHELD)	▪ Non-clonal cell pool materials at 2000-L scale for first-in-human clinical studies ▪ Abbreviated fed-batch process for pool selection ▪ Accelerated clone selection in Ambr® 15 system ▪ Clone selection to match pool product quality attributes ▪ No cell line stability study ▪ Leveraging prior process and manufacturing knowledge ▪ Analytical comparability study between DSs produced from MCBs of pool and clone	[9, 18]
Boehringer Ingelheim	▪ Production of GMP-grade drug substance in less than 3 months after transfection ▪ Initiation of Phase I clinical trial in 6 months ▪ Clonal cell line for commercial manufacturing in 8 months	▪ Transposase-based semi-targeted transgene integration ▪ Non-clonal cell pool materials at 2000-L scale for first-in-human clinical studies ▪ Selection of clonal cell lines with improved productivity and growth while maintaining highly comparable product quality profiles ▪ Clonal cell materials at 12 000-L scale for commercial process	[19]
Bristol Myers Squibb	▪ Shortened early-stage CMC development to approximately 6 months for 2 COVID-19 mAbs ▪ Completed late-stage cell culture PC within 4 months in comparison to a standard 1-year timeline	▪ Non-clonal cell pool materials for toxicological and first-in-human clinical studies ▪ Using RCB instead of MCB for commercial process development ▪ Using an MCB instead of a working cell bank (WCB) for PC studies and process performance qualification campaign ▪ Leveraging prior process and manufacturing knowledge ▪ Agile and rolling strategies for PC and in-process control report writing	[20]
Catalent	▪ Took less than 25 days for generation of pool RCBs ▪ Additional 12 weeks for clone banking and characterization	▪ Retroviral vector-based transgene integration (transduction) for generation of stable pools and clones	[21]
Junshi (TopAlliance) Biosciences	▪ DNA to IND in 4 months (etesevimab/JS016/LY-CoV016)	▪ Transient expression materials at 200-L scale to support preclinical, IND-enabling toxicology research and early CMC development ▪ Non-clonal cell pool (minipool) materials at 2000-L scale for first-in-human clinical studies ▪ Clonal cell material at 2000-L scale for late-stage and pivotal clinical trials	[22]
Lilly	▪ Transfection to first-in-human clinical trial in less than 2 months (bamlanivimab) in contrast to a traditional 17-month timeline	▪ Use of non-clonal cell pools through phase II clinical studies ▪ Use of mobile DP suite in DS manufacturing site ▪ Parallel technology transfers to multiple DS and DP sites.	[9]
Merck KGaA	▪ Production of clinical trial material within 4.5 months	▪ Transposase-based semi-targeted transgene integration ▪ Non-clonal cell pool materials at 200-L scale for preclinical safety studies and formulation development ▪ Non-clonal cell pool materials at 2000-L scale for first-in-human clinical studies ▪ Rapid RCB safety test using PCR- and NGS-based viral safety testing	[23]
Regeneron/Genentech	▪ Lead selection to initiation of clinical trials in 56 days (casirivimab and imdevimab) ▪ Development timeline shortened from approximately 12 to less than 6 months ▪ Acquiring EUA in less than 10 months	▪ Leveraging prior process and manufacturing knowledge ▪ Parallel and efficient technology transfer	[24]
WuXi Biologics	▪ DNA to IND in 3 months using non-clonal cell pool ▪ DNA to IND in 6 months using clonal cell line	▪ Non-clonal cell pool material at 200–2000-L scales for first-in-human clinical studies ▪ Clone selection to match pool product quality attributes ▪ Accelerated cell line stability study ▪ NGS-based viral detection method for MCB conditional release ▪ Leveraging prior process and manufacturing knowledge	[7, 8]

With regard to accelerating the selection of final clonal cell line in COVID-19 programs, synchronized execution of a simplified, phenotypic cell line stability study is the most impactful on the critical path to master cell bank (MCB) creation and overall IND timeliness [6–8]. To mimic seed train during actual manufacturing, conventional cell line stability study entails cell passaging, for example, from pre-master cell bank (PCB) for at least 60 population doubling levels (PDLs), after which productivities of the PCB and 60-PDL cells are evaluated. Depending on the doubling time of the cell line, the entire process can take about 3 months. For COVID-19 programs, instead of using a 60-PDL strategy, at WuXi Biologics, we performed stability study as early as permissible using clonal candidates passaged in the absence of selection pressure for 30 PDLs [6–8]. Coupled with high-throughput Ambr® bioreactor system during clone fed-batch screening to ensure scalability, the final clone can be selected as early as 10 weeks after transfection for MCB creation, which enables IND application in 6 months [7, 8]. To further mitigate the risk associated with cell line stability, single-cell qPCR can be used as an option to evaluate genetic stability of top clones in support of the accelerated cell line stability study [8, 25]. As an alternative, cell line stability study can also be taken off the critical path altogether with calculated business risk [18, 26]. In particular, Handlogten *et al.* described the speedy manufacturing of tixagevimab & cilgavimab from clonal cell lines without the typical evaluations of cell line stability. Of note, the speed to clinics was considered to outweigh potential risks of increased cost of goods manufactured (COG) that might result from a cell line with low population doubling level limit that could impede the scalability to larger manufacturing scales [18]. Furthermore, advanced expression technology such as targeted integration may eventually obsolete the cell line stability study for all biologics development [14, 27–30].

Following MCB creation, the next milestone on critical path to IND is GMP production of drug substance. Prior to release of MCB into a GMP production facility, conventional industry practice requires biosafety tests to be performed on MCB to prevent potential cross-contamination, which will instigate costly and time-consuming efforts in decontamination, investigation and corrective action. In accordance with industry practice, establishing negative results in sterility, mycoplasma and 28-day *in vitro* virus (IVV) testing is by default the acceptance criteria for MCB entry into a GMP facility at WuXi Biologics. Among these tests, IVV testing generally takes 7–8 weeks to complete, representing a major timeline holdup. To overcome this hurdle, we have devised a next-generation sequencing (NGS)-based viral detection method to enable the conditional release of MCB into a GMP facility in around 2 weeks, greatly shortening the turnaround time [7, 8]. Similarly, Agostinetto *et al*. have reported rapid safety testing on RCBs prior to entry into GMP facility using PCR- and NGS-based viral testing methods to eliminate risk of adventitious contamination [23].

Another important feature enabling the acceleration of CMC timeline is the leverage of proven platform technologies and processes to minimize the lead times of various process development and manufacturing activities. Process development activities for biologics generally include upstream, downstream, analytical method and formulation development, while manufacturing activities commonly encompass non-GMP pilot production, GMP drug substance production and GMP drug product production and filling. The use of proven platform technologies can shorten these activities into simplistic performance and feasibility checks, without multiple rounds of sequential workflows typically performed in a traditional CMC program. For instance, Regeneron emphasized the importance of prior knowledge and platform aspects in upstream and downstream processes to achieve rapid upstream process transfers and optimization of downstream purification steps of casirivimab and imdevimab [24]. Leveraging prior downstream knowledge, screening of chromatographical process parameters were performed within a development window of only several days between the first research batch and the toxicological batch. The toxicological batch was subsequently used as the only downstream process verification batch before the production of first-in-human clinical material. Bristol Myers Squibb also leveraged platform media and process using an RCB right after lead clone selection to compress timeline for late-stage process development by 2–3 months [20]. In addition, an MCB instead of a working cell bank (WCB) was used for process characterization (PC) studies and process performance qualification campaign to further fast-forward late stage development. At WuXi Biologics, we have also utilized platform knowledge in upstream, downstream, analytical and formulation processes, as well as manufacturing experiences including in process control strategies and raw material supplies, to ensure rapid execution of multiple COVID-19 programs [31].

## USE OF ACCELERATION STRATEGIES IN NON-COVID-19 PROGRAMS

The successful acceleration of COVID-19 programs is an outcome of convergence and consolidation of multiple technological and strategical innovations, as well as close collaboration of partnering companies. Apart from the use of non-clonal material for first-in-human clinical trials, which is unprecedented prior to COVID-19, many aforementioned technologies and strategies have been more or less applied in the industry, and over the years, CMC timeline has generally been reduced from around 18 to 12 months. However, the successful demonstrations of CMC acceleration under COVID-19 have urged the industry to rethink whether these acceleration approaches can become a new modus operandi and further reduce regular CMC timeline.

**Figure 1:**
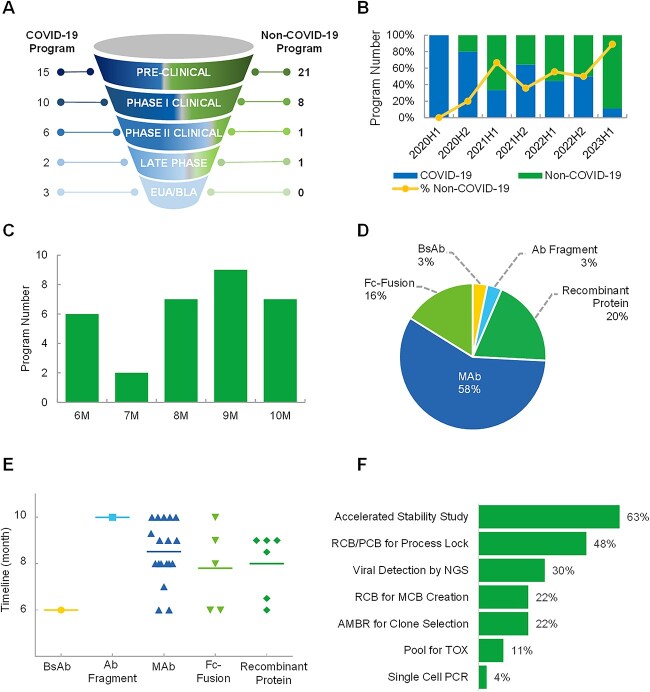
CMC programs with a DNA-to-IND timeline of ≤10 months at WuXi Biologics between January 2020 and June 2023. (A) Numbers of COVID-19 and non-COVID-19 programs at different clinical stages. (B) Percentages of COVID-19 and non-COVID-19 programs according to program kick-off date. (C) Timeline distribution of Non-COVID-19 programs. (D) Molecule types of non-COVID-19 programs. (E) Timeline distribution of non-COVID-19 programs according to molecule type. (F) Primary acceleration strategies used in non-COVID-19 programs. Note: Only integrated programs, i.e. programs covering the whole CMC process from cell line development to drug product filling (DNA to IND), are included. This criterion excluded transfer-in and transfer-out programs, which may have a timeline of ≤10 months at WuXi Biologics but are not actually representative of the application of acceleration strategies intended for this analysis. Ab, antibody; BsAb, bispecific antibody; IND, investigational new drug; MAb, monoclonal antibody; PCB, pre-master cell bank; MCB, master cell bank; NGS, next-generation sequencing; RCB, research cell bank; TOX, toxicological study; PCR, polymerase chain reaction.

As a leading global biologics contract research, development and manufacturing organization, WuXi Biologics regularly receives requests from clients concerning expediting CMC readiness to meet aggressive IND application timelines. To have a sample of the global CMC acceleration trend, we analyzed CMC programs performed at WuXi Biologics from January 2020 to June 2023 with an intended DNA to IND timeline of ≤10 months, which is considered aggressive by industry standard. As shown in Fig. 1A, we categorize the programs into COVID-19 and non-COVID-19 at different clinical stages, namely, preclinical, phase I, phase II, late phase and EUA/BLA. As of June 2023, three COVID-19 mAbs developed at WuXi Biologics had been granted EUA status. Thirty-three other neutralizing COVID-19 biologics are at preclinical or different clinical stages. For non-COVID-19 programs, the number of programs with a CMC timeline of ≤10 months is 31, with the majority of the programs at preclinical or phase I clinical stage. From the perspective of program initiation date, we observe a downward trend of COVID-19 program numbers and correspondingly an upward trend of non-COVID-19 program numbers from January 2020 to June 2023. This is in accordance with the progression of the pandemic, with the pandemic peaking during 2020–2 and subsequently most countries transitioning away from an emergency status to a general public health priority status. With the continuous conquest of shortening drug development timeline to swiftly supply therapeutics to patients worldwide, we anticipate a continuation of the upward trend for non-COVID-19 programs.

To gain more insights on the features of non-COVID-19 programs, we further analyzed the timeline, molecule type and acceleration strategy of the programs as illustrated in Fig. 1C–F. As shown in Fig. 1C, accelerated non-COVID-19 programs have timelines of between 6 and 10 months. Unlike COVID-19 programs where non-clonal stable pool can be used for material generation for phase I clinical trials upon authorization from regulatory agency, non-COVID-19 programs follow the traditional requirement of generating a clonal cell line, thus demanding a longer CMC duration for the initiation of first-in-human clinical trials. The molecule types of the accelerated non-COVID-19 programs are depicted in Fig. 1D. Almost 60% of the programs are mAbs. This is followed by recombinant protein (20%) and Fc fusion protein (16%). Bispecific antibody (BsAb) and other antibody (Ab) fragments constitute 3% each of the total number. One reason mAb still represents the most frequently accelerated molecule type is the rich prior knowledge for mAb. With an increasing complexity of the molecule format, the need for process development and optimization could increase proportionately. However, with a robust platform and broad development experiences, acceleration of different modalities with complex formats is not at all unattainable. This is clear from Fig. 1E, in which a 6-month CMC strategy has been applied to almost all molecule types. In terms of acceleration strategies (Fig. 1E), use of accelerated cell line stability study [7, 8] and RCB/PCB instead of MCB for process lock are the two most frequently used methods for timeline reduction. Application of NGS-based viral testing for MCB conditional release [7, 8, 23] also constitute 30% of the overall application. Additionally, a number of projects employed Ambr® bioreactors for clone screening [8], pool for toxicological study [10–16] and single-cell PCR [8, 25] for timeline acceleration.

With regard to applying the acceleration strategies on molecules of different modalities with complex formats, an assessment of molecule developability using small-scale transient or pool materials before cell line development is highly recommended [32, 33]. Developability assessment can provide predictive and useful information on product biochemical features, which can assist in determining whether the use of acceleration strategies is suitable, or a more traditional approach is required to avoid potential developability hurdles. Depending on molecule complexity and prior data, developability studies can be designed in a fit-for-purpose manner [32, 33] and with middle- to high-throughput formats when combined with automated laboratory configurations [34–37]. At WuXi Biologics, we routinely perform chain ratio studies for BsAbs using transient expression system in 24- or 96-deep well plate formats to gauge the best ratios for two or multiple chains before proceeding to stable transfection. Additionally, different codons, signal sequences and/or various vector elements can also be screened to obtain optimized combinations that indicate better productivity and product quality for a molecule of interest with a complex format [32].

## A DEMONSTRATED 6-MONTH DNA-TO-IND TIMELINE FOR BIOLOGICS DEVELOPMENT

**Figure 2:**
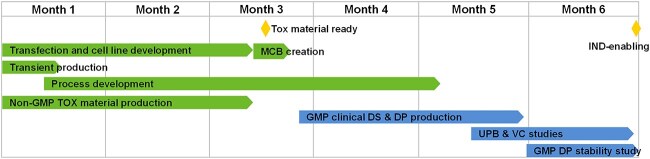
An accelerated 6-month CMC timeline proposed for general biologics development. DP, drug product; DS, drug substance; GMP, good manufacturing practice; IND, investigational new drug application; MCB, master cell bank; Tox, toxicology study; UPB, unprocessed bulk; VC, viral clearance.

Combining prior knowledge and experiences from developing COVID-19 mAbs, it is clear that a 6-month DNA-to-IND timeline for biologics can be routinely executed. In Fig. 2, we outline an overall CMC workflow involved in such a strategy. The initiation step of the program is the simultaneous implementations of transient expression for material generation and stable transfection for cell line development. As shown in Table 1, transient materials have been used by Xu *et al.* at a 200-L bioreactor production scale to support preclinical, IND-enabling toxicology research and early CMC development of the neutralizing COVID-19 mAb etesevimab (JS-016), demonstrating the feasibility of this strategy [22]. For the proposed generic 6-month accelerated DNA-to-IND timeline, transient expression can be performed at different production scales based on estimated material need and production yield. Materials generated from transient production are intended to be used for (1) platform formulation evaluation, (2) formulation development and (3) assay development. It should be noted that a liquid or frozen liquid formulation, but not lyophilized product, is assumed. In parallel with transient production, accelerated cell line development with a timeline of 2.5 months from DNA to final clone identification is initiated. A detailed description of the accelerated cell line development can be found in our previous publications [7, 8].

Initial cell culture (upstream) process development overlaps cell line development, for example, by applying high-throughput Ambr® 250 bioreactor system for top clone screening and selection [7, 8]. After the final clonal candidate has been selected, MCB creation can be initiated. Of note, the use of NGS-based adventitious viral testing renders the MCB release off the critical path to allows the accelerated GMP drug substance (DS) manufacturing using MCB (Fig. 2). One further round of intensive and accelerated upstream development and optimization can be performed for the final clonal cell line, typically using 3-L bioreactors. For the purification process, a platform process evaluation is firstly performed, which is followed by one round of intensive and accelerated process development, typically assuming one clarification step, an intermediate-depth filtration step, up to three column steps, a virus inactivation step, a viral filtration step and a final ultrafiltration/diafiltration (UF/DF) step. Again, accumulated process knowledge with a variety of molecules is key to the success of process development acceleration, especially for complex recombinant proteins. Finally, a process confirmation run is performed using the RCB of the final clonal cell line with the purpose of verifying the upstream, downstream and formulation processes, as well as providing material for assay qualification.

A batch of non-GMP DS manufacturing, typically at 200-L scale, is performed using stable pool for toxicological material generation, i.e. the “pool-for-tox” strategy is employed. Material generated in this non-GMP batch is also used for reference standard preparations and testing, drug product (DP) engineering run and engineering DS/DP stability studies. Owing to timeline constraint, platform upstream and downstream processes, including the use of non-dedicated resins, are expected to be applied. To reduce the risk of large-scale manufacturing, we routinely inoculate a satellite 3-L bioreactor a number of days prior to the large-scale bioreactor to preemptively detect any issue that might occur in the large-scale production. For the GMP manufacturing of DS, acceleration can be achieved by efficient technology transfer and the use of platform raw materials. In addition, the unprocessed bulk testing and viral clearance testing are accelerated to less than 2 months as compared with a typical 4 months turnaround time for each (Fig. 2).

Acceleration of cell line development and overall CMC activities may sometimes make some of the ensuing activities, such as GLP toxicology studies, become rate-limiting. In such cases, effective planning and coordination between CMC and preclinical toxicology teams become crucial. During COVID-19, a minimum preclinical data package required to move a candidate therapeutic into clinical trial to support rapid development of COVID-19 therapeutics has been proposed [38]. Interestingly, Baldrick reported that the number and types of preclinical studies were not reduced for neutralizing COVID-19 mAbs that had been granted EUA or CMA status by regulatory agencies [39]. The main reason for the rapid authorization of neutralizing COVID-19 mAbs is concluded to be the recognition by regulatory agencies that mAbs can specifically target the receptor binding domain (RBD) of the spike protein leading to neutralization of SARS-CoV-2 [39].

## CONCLUSION

Under the COVID-19 pandemic, development timeline and IND application readiness have undergone major acceleration for neutralizing COVID-19 mAbs. The expedited speed to clinic without compromising product quality and safety is a result of convergence and consolidation of innovative technologies and CMC strategies in biologics development over the years, as well as close collaboration of pharmaceutical industry and regulatory agencies. With the emergency status of COVID-19 closing out, many innovations used in COVID-19 programs are expected to continue to impact future biologics development. We are of the view that these innovative approaches can be widely applied in all disease areas to reduce overall biologics development timeline for greater speed to clinic. The 6-month CMC timeline we propose here represents an exemplar that can be used as a framework for CMC program planning and management. With continuous technological innovation, efficacious innovative biologics are expected to reach patients in need at a greater pace.

## Data Availability

The data underlying this article will be shared on reasonable request to the corresponding author.
